# Estimates of the stochasticity of droplet dispersion by a cough

**DOI:** 10.1063/5.0070528

**Published:** 2021-11-23

**Authors:** Shrey Trivedi, Savvas Gkantonas, Léo C. C. Mesquita, Salvatore Iavarone, Pedro M. de Oliveira, Epaminondas Mastorakos

**Affiliations:** Department of Engineering, University of Cambridge, Trumpington Street, Cambridge CB2 1PZ, United Kingdom

## Abstract

In this paper, the statistical distributions of the position and the size of the evaporating droplets after a cough are evaluated, thus characterizing the inherent stochasticity of respiratory releases due to turbulence. For that, ten independent realizations of a cough with realistic initial conditions and in a room at 20 °C and 40% relative humidity were performed with large eddy simulations and Lagrangian tracking of the liquid phase. It was found that although turbulence decreases far from the emitter, it results in large variations in the spatial distribution of the droplets. The total suspended liquid mass after 60 s from the cough is in good agreement with that estimated by a one-dimensional model accounting for settling and evaporation under quiescent conditions, while deposition times of droplets in the 10–100 *μ*m range are found to vary significantly, reflected in the mass of liquid, and hence the virus content, potentially inhaled by a receptor. The high variability between events is due to the local fluctuations of temperature, humidity, and velocity on droplet evaporation and motion. The droplet distribution suggests that, in the absence of face coverings, an unprotected cough is not safe at 2 m away from the emitter even outdoors. The results indicate that mitigation measures, such as ventilation to address long-range transmission, can be based on the total suspended liquid content evaluated from reduced-order models. However, the large variability of viral content in the near field produces wide variations in estimates of risk; therefore, a stochastic approach is needed for evaluating short-range transmission risk.

## I. INTRODUCTION

The ongoing pandemic caused by SARS-CoV-2 (COVID-19) has reinforced the need to better understand the fluid mechanics controlling the spread of airborne diseases. Despite strict global measures to mitigate the spread of the COVID-19 disease, its contagion has been unprecedented.^1^ This may be attributed at least in part to the limited knowledge at the start of the pandemic about the spread of droplets/aerosols that can carry the pathogens over long distances.^2,3^ Efforts to improve the understanding of the spread of such diseases^4^ and to develop models that can better predict infections are underway.^5–10^

The exhaled flow contains pathogen-carrying droplets of varying sizes, and their trajectory is governed by their initial size, the influence of gravity, the local and ambient temperatures and relative humidity, and the gas velocities. The small droplets can stay suspended in the air for a long time and can carry the pathogens over significantly long distances, whereas the larger droplets follow a ballistic trajectory and tend to settle down quickly under the influence of gravity.^11^ The distinction between large ballistic droplets and small droplets is usually assumed to be ∼100 *μ*m, while the cutoff for droplets that remain suspended in air for long times is typically considered as 10 *μ*m,^12^ although it is still inconclusive whether that is the case.^13^

Early measurements to capture droplet size and spread^14^ used collection media, such as slides. These were limited by the lowest resolution of the droplets, and they usually captured droplets of super micrometer sizes. However, it was reported at the time that sub-micrometer droplets were also very likely. Later, optical-based counters^15,16^ reported the dominance of submicrometer droplets. Recent studies^17–20^ used more advanced methods to capture the droplet size distribution exhaled from respiratory events, such as coughing. The size distribution of the droplets and the flow rates for a cough were well characterized at the source, i.e., the mouth, by Johnson *et al.*^20^ and Gupta *et al.*,^21^ respectively. The droplets reach an equilibrium size that can be 20%–40% of the initial droplet size, depending on the ambient conditions or the composition of the saliva.^5,22–24^

Bourouiba *et al.*^2^ performed experiments and theoretical analyses to characterize the flow from violent respiratory events, such as coughing and sneezing. In such events, a jet of air of limited duration containing respiratory droplets is exhaled, forming a turbulent puff that remains suspended in the air.^2,3^ The local conditions within the turbulent puff act to extend the evaporation time of the exhaled droplets.^3,9^ In subsequent direct numerical simulation (DNS) analyses, the ambient relative humidity was also found to significantly increase the droplet evaporation time,^25–27^ especially those with a diameter below 30 *μ*m.^27^ Rosti *et al.*^26^ found that turbulence increases the lifetime of the droplets, and an underestimation of 100% in droplet evaporation time was reported when the turbulence effects were filtered out. Although reasonable estimates of the horizontal displacement of the exhaled puff can be obtained from reduced-order models,^28,29^ gas-phase only DNS of a cough^30^ has shown that a large deviation from the predicted values could arise due to difficulties in predicting jet-to-puff transition effects and puff topology in such models, in addition to turbulence itself as discussed previously. Still, despite the in-depth physical insight obtained from DNS concerning small-scale interactions between liquid and gas phases, its significant computational cost hinders both the evaluation of long events and the quantification of event-to-event variations.

Concerning the spread of droplets in a respiratory release, works carried out using Reynolds-averaged numerical simulations^31–33^ indicate that droplets, especially those of intermediate size, seem to be contained within 2 m from the infectious individual following a cough at stagnant conditions, while typical outdoor wind speeds can triple their horizontal reach, especially those of intermediate sizes 50–100 *μ*m. More recently, large eddy simulations (LES) of coughs and sneezes^34,35^ have been performed to extract various quantities of interest, accounting for turbulence-induced effects. Liu *et al.*^34^ showed through six LES realizations that global puff properties, such as its centroid, volume, momentum, and buoyancy, do not vary significantly from event to event. The need of a significant number of realizations has been stressed as of utmost importance to fully provide information regarding turbulence properties of respiratory releases.^30,34^ Despite reports on the impacts of turbulence on the maximum reach and fall-out of droplets,^34,35^ a detailed quantification of their statistical distribution is still under development.

Mathematical models of host-to-host droplet transmissions for physical distancing measures were studied in several works.^6,9,36–38^ Overall, the results from these studies generally concluded that 2-m guidelines are only effective as long as other measures, such as masks, are being utilized. CFD (computational fluid dynamics) studies performed in an indoor environment^27,31,32,39,40^ and outdoors^33^ found similar conclusions regarding the physical distancing measures. The effect of masks on disease transmission was analyzed in several studies^32,41,42^ concluding that masks can cut the droplet transmission distance significantly by suppressing the exhaled flow as well as altering the size distribution of the exhaled droplets. As mentioned, the presence of wind was also seen to assist the exhaled flow and consequently increase the distances over which the infection can be transmitted both with or without a mask.^36,43^

The importance of the local conditions within the turbulent puff, ambient conditions, and turbulence on droplet evaporation time has been discussed in several studies.^44,45^ The turbulent flows associated with events, such as a cough, are inherently stochastic, which may cause variation of two-phase flow parameters, such as the physical location of the droplets of different sizes and their concentration. Recent host-to-host infection models, such as those mentioned previously, provide an average estimation of where the droplets are and the effect of physical distancing measures on this estimation. However, for diseases which contagion may occur due to inhalation of only a few virions, ignoring the effect of turbulence and its effects as high spatial and event-to-event variations in respiratory releases may significantly impact the evaluation of the infection risk. In the context of disease transmission at population level, where contact and transmission rates between individuals, among other factors, are used in probabilistic models to estimate the evolution and spread of an epidemic, accounting for the stochastic nature of respiratory releases becomes even more relevant. To the best of the authors' knowledge, the stochasticity of such respiratory flows, including the cough, has not been quantified yet.

In this work, high-fidelity large eddy simulation (LES) is employed to simulate the gas flow exhaled in several independent cough events in a stagnant environment. Lagrangian droplet tracking is used to evaluate the combined motion and unsteady evaporation of droplets of various sizes, characteristic of a cough, as they are ejected with the turbulent gas puff. Ten realizations were performed in an ambient setting of 20 °C and relative humidity of 40% with the objective of examining the flow-driven stochasticity of parameters relevant to disease transmission in the presence of buoyancy and with significant evaporation of the respiratory droplets due to the entrainment of air with low relative humidity by the gas puff. The parameters evaluated include the suspended liquid mass, the size, and spatial distribution of the droplets, as well as the number of virus copies that can be inhaled by a receptor at a specific horizontal distance from an infectious person. The results of the simulations are then put in context of short-range transmission, where the risk of infection is evaluated at different horizontal distances from the infectious individual to illustrate the potential impact of such flow fluctuations on mitigation measures, such as physical distancing.

The remainder of the paper is structured as follows. In Sec. II, the methodology used for this analysis is discussed. This includes the LES models, the Lagrangian droplet tracking technique, and the models for evaporation. Next, the results from the gaseous flow obtained from the LES and those from the tracking analysis of the droplets are provided and then discussed in the context of disease transmission. In Sec. V, the key conclusions are summarized, and improvements for better distancing and ventilation measures are discussed.

## II. METHODOLOGY AND SIMULATION SETUP

### A. Models

The large eddy simulation (LES) of the cough is carried out using the software CONVERGE. The governing equations for LES are readily available in the literature and hence are not presented here (e.g., see Ferziger and Perić^46^). In this work, the sub-grid scales are modeled using the Dynamic Smagorinsky model based on the eddy viscosity approach.^46^ A finite-volume second-order accurate spatial scheme coupled with pressure implicit with splitting of operator (PISO) iterative algorithm^47^ and an implicit first-order temporal scheme is employed for solving the governing equations of the flow.

The motion and evaporation of droplets are calculated *a posteriori* with an unsteady in-house Lagrangian tracking code, which uses the instantaneous gas-phase flow field solved by LES to produce an accurate time evolution of droplets trajectory and properties. The motion of the *i*th droplet defined by the instantaneous location $x_{i}$, velocity $v_{i}$, mass *m_i_*, and temperature *T_i_* is solved using the following equations:^48,49^

$$\frac{dx_{i}}{dt}=v_{i},$$ (1)

$$\begin{array}{c} \frac{dv_{i}}{dt}=\frac{3C_{D,i}}{4d_{i}}\left(\frac{\rho }{\rho_{l}}\right)|u+u_{i}^{\prime }-v_{i}|u+u_{i}^{\prime }-v_{i})\\ +\left(1-\frac{\rho }{\rho_{l}}\right)g, \end{array}$$ (2)

$$\frac{dm_{i}}{dt}=\pi d_{i}\rho D_{g}{\text{Sh}}^{*}\;\text{ln}\;(1+B_{M}),$$ (3)

$$\frac{dT_{i}}{dt}=\frac{{\dot{m}}_{i}}{m_{i}c_{p,l}}\left(h_{v,l}-\frac{c_{p,v}(T_{g}-T_{i})}{B_{T}}\right),$$ (4)

where $C_{D,i}$ is the drag coefficient, *d_i_* is the diameter of the *i*th droplet, *ρ* and *ρ_l_* are the density of gas-phase and liquid-phase (i.e., droplets), respectively, **u** is the velocity vector of the gas, $u_{i}^{\prime }$ is the vector of the velocity fluctuations, **g** is the acceleration due to gravity, *D_g_* is the mass diffusion coefficient, ${\text{Sh}}^{*}$ is the modified Sherwood number, $B_{M}=(y_{w,\infty }-y_{w,s})/(y_{w,s}-1)$ and $B_{T}=m_{i}c_{p,l}(T_{\infty }-T_{i})/Q_{g}$ are the Spalding mass and heat transfer numbers, ${\dot{m}}_{i}=dm_{i}/dt,\;c_{p,l}$ and *c_p_* are the specific heat of water in liquid and vapor phase, respectively, $h_{v,l}$ is the latent heat of vaporization, $y_{w,\infty }$ and $y_{w,s}$ are the water mass fractions at the droplet surroundings and at the droplet surface, and *Q_g_* is the heat flux.

In LES, the fluctuating part of the gas-phase velocity is accounted for directly by the velocity vector provided by the resolved flow. Although the sub-grid random component could be included,^50^ these are ignored here since the grid size remains small in the region of interest and hence the flow is reasonably well-resolved, as will be discussed in Sec. II B. The drag coefficient $C_{D,i}$ in the aerodynamic drag term of Eq. (2) is calculated using the Schiller–Naumann correlation.^51^ It is a function of the Reynolds number of the droplet, i.e., ${\text{Re}}_{i}=\rho d_{i}|u+u_{i}^{\prime }-v_{i}|/\mu$, where *μ* is the dynamic viscosity of the gas phase. The Reynolds number ${\text{Re}}_{i}$ is calculated using the relative velocity between the particle and the carrier phase. The heat and mass transfer between the droplet and the surrounding gas, considered in Eqs. (3) and (4), accounts for the effect of Stefan flow due to evaporation. Therefore, a modified Sherwood number is used, defined as ${\text{Sh}}^{*}=2+({\text{Sh}}_{0}-2)/F_{M}$, where ${\text{Sh}}_{0}$ is the actual Sherwood number obtained with the widely used Frossling's correlation,^52^ and then corrected for the film thickness of the surrounding gas by the correction factor *F_M_* proposed by Abramzon and Sirignano.^53^

In the present calculations, the local moisture in the air is used in the calculation of the evaporation rate, through the mass fraction of water vapor in the definition of the Spalding number *B_M_*. The volume fraction of water vapor in the ambient air, $x_{w,a}$, is related to the relative humidity RH by $x_{w,a}=\text{RH}\;p_{w,\mathrm{sat}}(T_{a})/p_{a}$, where $p_{w,\mathrm{sat}}$ is the water saturation pressure and *T_a_* and *p_a_* are the ambient temperature and pressure, respectively. The volume fraction of water vapor *x_w_* in the surroundings of a single droplet is calculated from the corresponding mass fraction of water vapor *y_w_*, which is estimated as $y_{w}=(1-\xi )y_{w,a}+\xi y_{w,m}$, where *ξ* is the mixture fraction at the droplet location and $y_{w,a}$ $y_{w,m}$ are the mass fraction of water vapor in the ambient air and in the mouth, respectively. The mixture fraction is a passive scalar defined to be unity in the undiluted exhaled flow and zero in the ambient air and is solved by a transport equation in the LES.

Although local conditions of the gas-phase puff are considered in the evaluation of the droplets' evaporation rates, the effect of droplet evaporation on the gas-phase field is neglected in this one-way coupling approach, as it is expected to be minimal due to the small mass loading of the liquid phase. Here, droplets are modeled as being pure water; however, evaporation is limited down to 6% of the initial droplet volume to mimic the presence of nonvolatile components in the saliva (as was done in Aliabadi *et al.*^54^). This approach results in a droplet equilibrium diameter equivalent to the one found for high-protein saliva by de Oliveira *et al.*^5^ and is a good approximation to represent saliva evaporation in the studied conditions. Finally, secondary breakup and coalescence of the droplets are neglected for the purposes of this study.

### B. CFD domain and boundary conditions

**FIG. 1.**
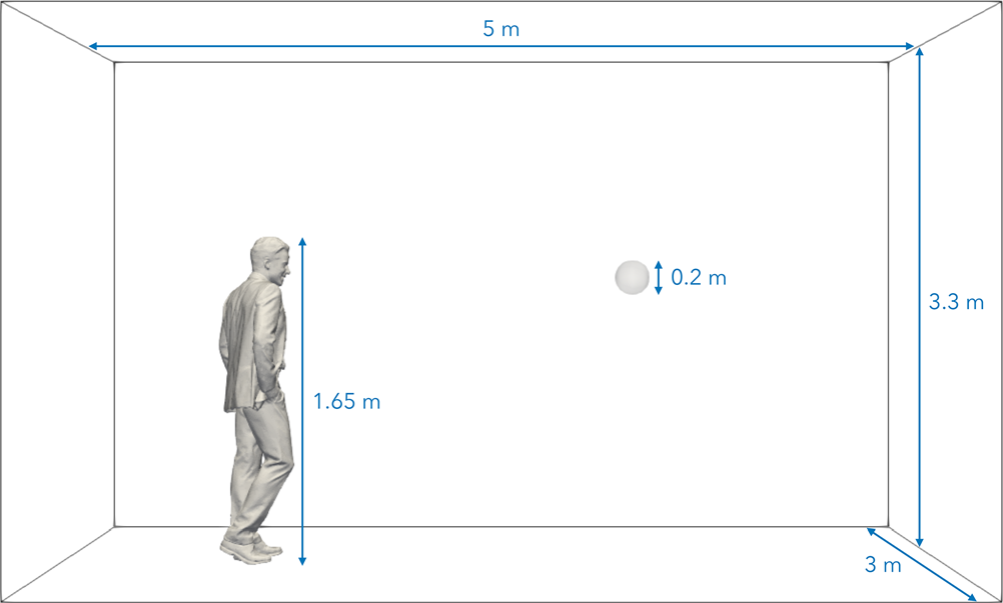
CFD domain with corresponding dimensions and indication of the breathing zone, i.e., a sphere of diameter of 0.2 m placed 2 m away from the emitter.

The simulation domain is shown in Fig. 1 and is composed of a cuboid room of dimensions 5 × 3 × 3.3 m^3^. In Fig. 1, the breathing zone of a possible receptor is also shown. To estimate the risk of infection by a receptor, a spherical probe of volume $V_{bz}=(\pi /6)d_{bz}^{3}$ is considered, where the subscript *bz* stands for breathing zone, and the chosen diameter is $d_{bz}=0.2$ m.^55^ The subject is 1.65 m tall and is placed on the left side of the room (see Fig. 1). The body of the subject is treated as a wall set at room temperature. The subject's mouth is set as an inflow with a net mass flow representative of a cough taken from Gupta *et al.*^21^ for a male subject. The subject's mouth has an area of ∼4 cm^2^ as in Gupta *et al.*^21^ The exhaled breath is set at standard human body temperature 309 K and at 100% relative humidity with a ${\text{CO}}_{2}$ composition of 0.07% in terms of mass.

The flow in the domain is nearly stagnant with a nominal initial velocity of 0.01 m/s set in the *x*-direction. There is no initial turbulence. The ambient temperature is set as 293 K and the relative humidity at 40%. The rest of the domain boundaries are as follows: the left boundary is set as inflow with airflow equal to the initialized domain velocity, i.e., 0.01 m/s set in the *x*-direction, the bottom boundary is treated as no-slip wall and all the rest of the boundaries are set as outflow.

At the start of the simulation, the gaseous cough flow is exhaled by the subject. A small change in the flow rate (<0.01% of the peak flow rate) was introduced for different realizations right at the beginning of the cough. The apparently random nature of turbulence^56^ ensures that even this small change results in a different turbulent flow field while still keeping the overall cough flow rate the same between the realizations. The peak flow rate of the cough occurs at 0.1 s after the start and then the flow gradually decreases. This peak mass flow rate is 5 l/s, and the corresponding peak velocity is 12.5 m/s. The Reynolds number $\text{Re}=u_{\mathrm{peak}}\ell_{\mathrm{mouth}}/\nu$ based on the peak velocity of the jet is estimated as ∼15 000, which is high enough to make the flow turbulent. An entire duration of a typical single cough is about 0.5 s,^21^ after which it spreads within the domain for 60 s. Lagrangian tracking of the emitted droplets is performed in post-processing. The size distribution and the concentration of the droplets in the exhaled gas are taken from Johnson *et al.,*^20^ with 5000 droplets injected at the start of the simulation, typical of a cough.^14^

One of the major characteristics of the CONVERGE code is that it auto-creates a cut-cell Cartesian mesh relying on an adaptive mesh refinement (AMR) strategy.^57^ This approach is particularly convenient for LES, as it ensures that the zone of interest of the flow is well refined (thus improving the resolution), while the mesh is coarsened elsewhere to reduce computational cost. For this case, this means that the mesh will remain sufficiently fine to ensure a good resolution within the cough puff, as it moves through the domain. The mesh refinement criteria were set as a minimum value of sub-grid velocity, mixture fraction and mass fraction of ${\text{CO}}_{2}$, with the minimum and maximum cell size being 3 and 50 mm, respectively.

## III. RESULTS

This section starts with a qualitative assessment of the gas flow exhaled in a cough and its spatial spread, validated by scaling laws and in comparison with experiments.^2^ Then, the motion of the exhaled droplets is presented, with focus on the stochasticity of their position due to the turbulent motion of the gas phase. The results given are then put in the context of physical distancing measures in Sec. IV, where the impact of flow-driven stochasticity is evaluated in terms of the variability of the risk of infection.

**FIG. 2.**
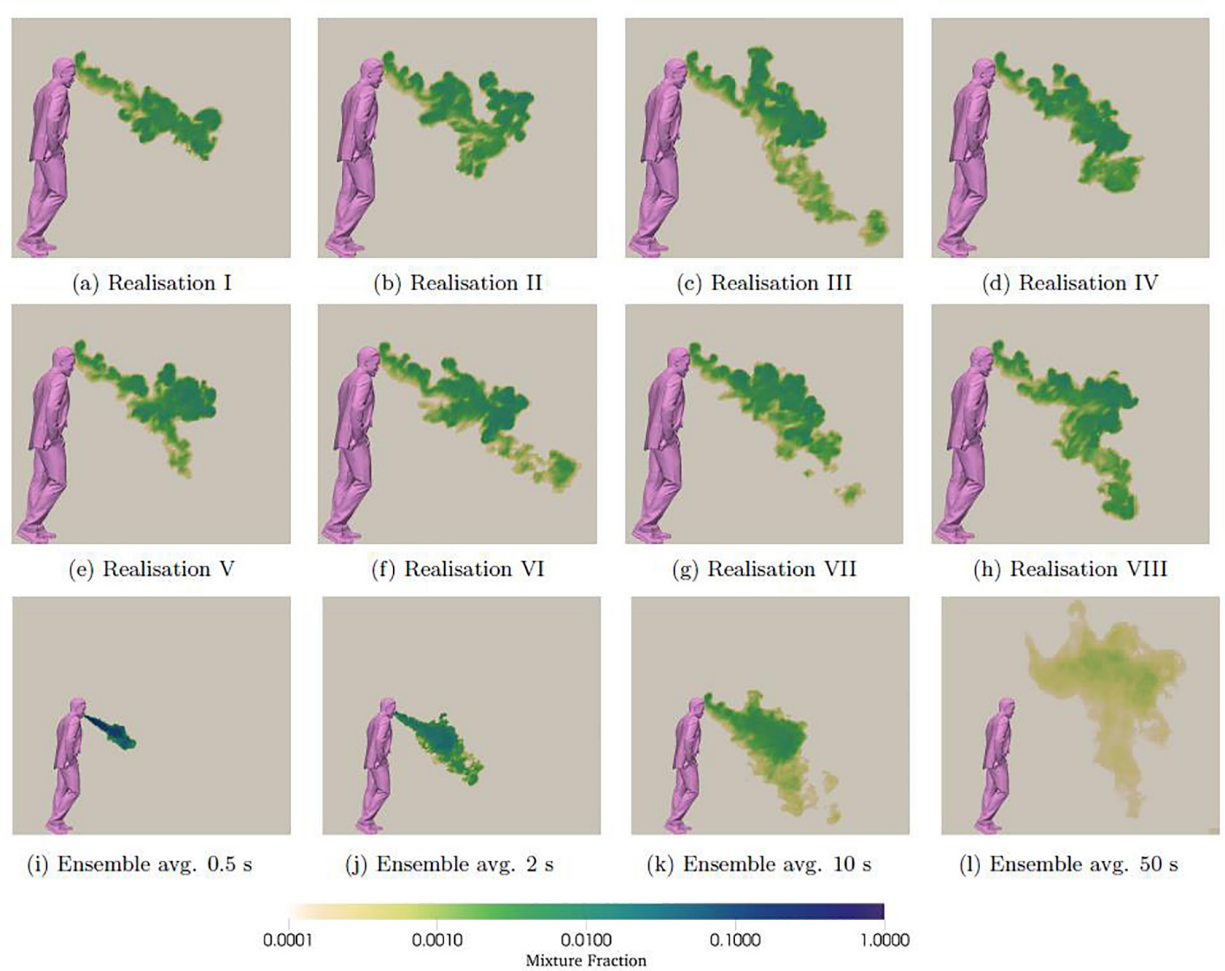
2D middle-plane slices of the mixture fraction in a cough: (a)–(h) instantaneous snapshots of eight different realizations of scalar distributions from a cough after 10 s of physical time. Significant differences in mixture fraction field can be observed for different realizations of the flow. (i)–(l) Mixture fraction distributions averaged over 10 different realizations, taken at (i) 0.5, (j) 2, (k) 10, and (l) 50 s.

The sudden ejection of the exhaled breath in a cough involves high velocities at the mouth and, as such, produces a turbulent flow, which means each cough is unique in terms of the motion fluid particles undergo. This can be seen in Fig. 2, which shows eight simulation realizations as 2D slices of the mixture fraction at the middle of the domain at 10 s after the cough. As expected, since this is a high-Reynolds number turbulent flow, each realization is different despite the overall similar pattern of spreading. The exhaled flow has two distinct phases:^2^ the initial phase during which the flow is exhaled like a turbulent jet, and a second phase when the exhaled jet becomes a turbulent puff of finite duration that grows by entraining air from the surroundings. These phases can be seen through the ensemble-averaged mixture fraction field of all realizations (Fig. 2) at 0.5 and 10 s, for instance.

**FIG. 3.**
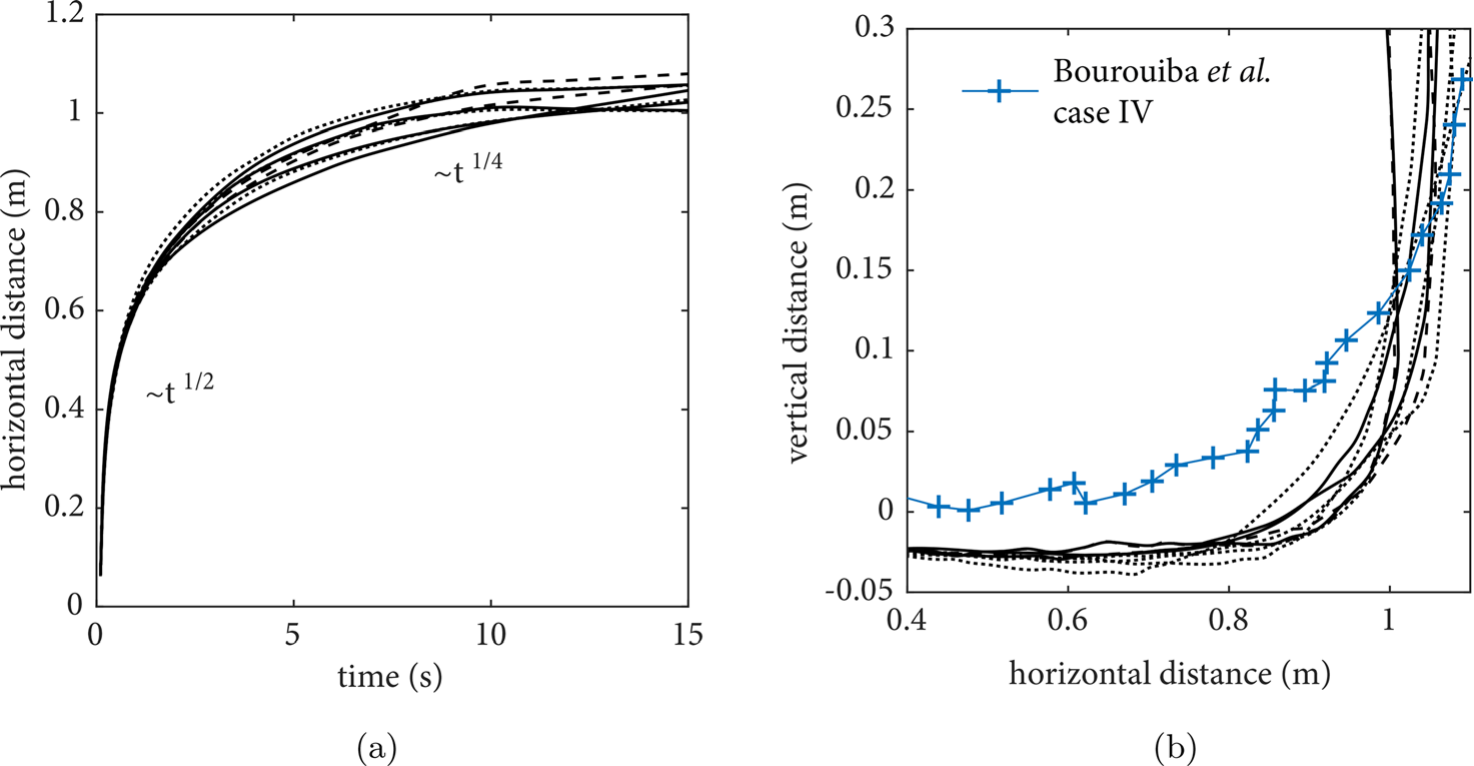
Scaling analysis of the turbulent puff centroid: (a) horizontal distance vs time and (b) horizontal distance vs vertical distance.

Figure 3(a) shows the evolution of the distance traveled by the centroid of the turbulent jet/puff *x* for each realization. Consistent with Bourouiba *et al.,*^2^ the initial jet phase follows $x∼t^{1/2}$ whereas the turbulent puff follows $x∼t^{1/4}$. The horizontal distance vs the vertical distance traveled by the centroid [Fig. 3(b)] exhibits the typical behavior of turbulent puffs moving under the influence of initial momentum and buoyancy found in experiments (case IV, Bourouiba *et al.*^2^). Due to the initial jet angle, the flow moves slightly downward until 1-m horizontal distance when buoyancy causes the flow to move upward, as described by Gupta *et al.*^21^ Thus, the results of the present LES follow the scaling laws and exhibit a qualitative agreement with trends observed in experiments.

**FIG. 4.**
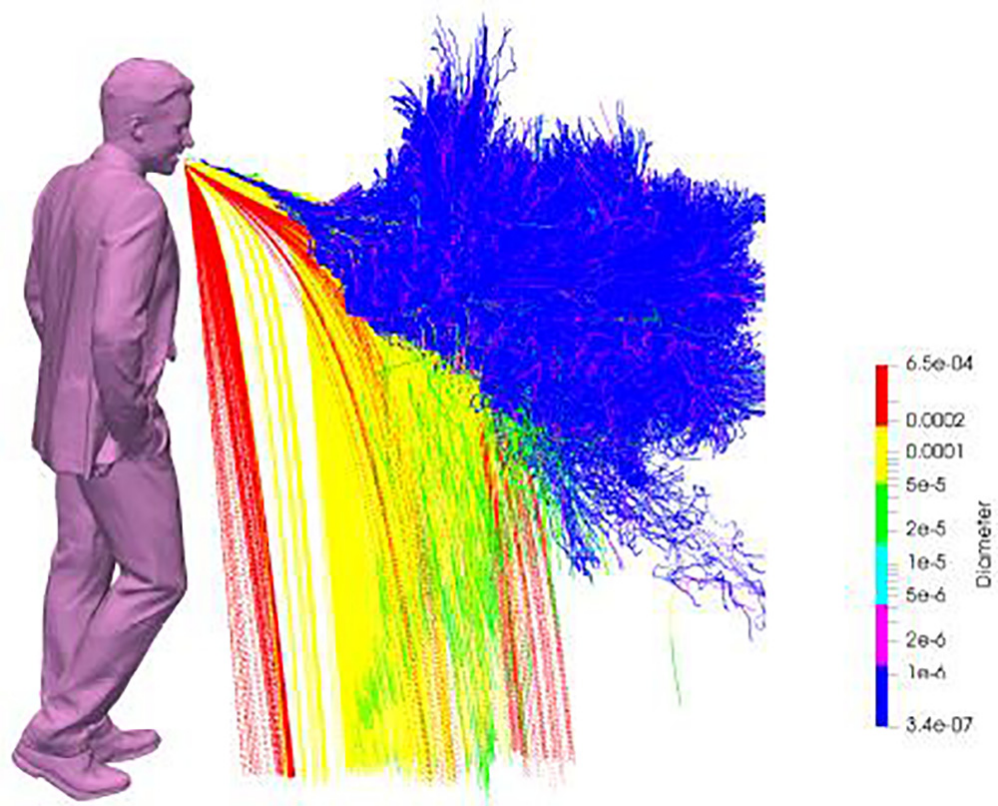
Time history of the emitted droplets, colored by diameter (in meters), during a cough, showing their trajectory in the interval 0–10 s. All ten realizations are overlapped, providing an ensemble of the droplets and their trajectories.

**FIG. 5.**
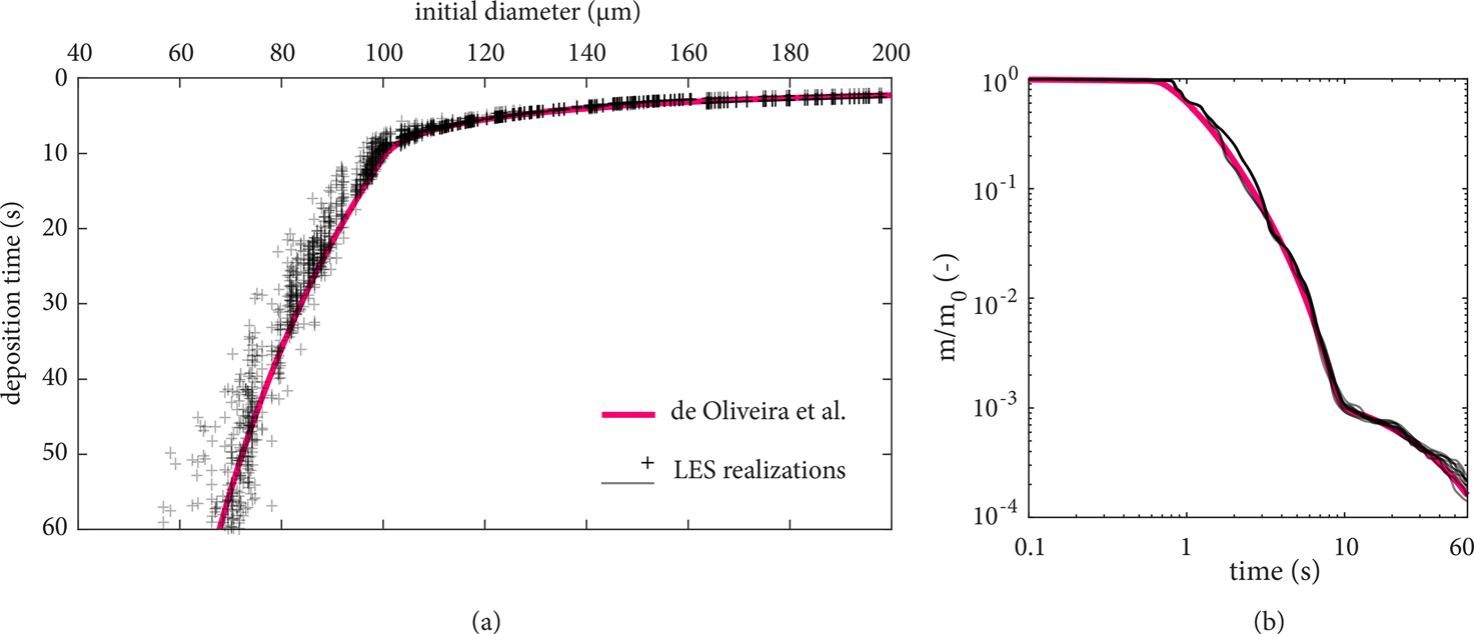
(a) Deposition time of droplets and (b) mass decay normalized in terms of the initial mass exhaled in all LES realizations. The results are compared with the results from the 1D model from de Oliveira *et al.*^5^ (solid red line). Significant variation in deposition times of droplets in the 10–100 *μ*m range leads to small variations of the total suspended mass of the cloud, as seen in the LES realizations. This is attributed to the effects of the local humidity, temperature, and turbulent motion within the turbulent puff surrounding the droplets.

While the results in Fig. 3 illustrate the motion and spread of the exhaled gas, pathogens are in fact transported by droplets, both evaporating and fully evaporated ones (known as droplet nuclei). Since the initial size distribution is not uniform, we may expect droplets to respond differently to the flow and under the action of gravity. This is shown in Fig. 4 through the trajectories of individual droplets up to 10 s. The fact that large droplets exhibit a ballistic behavior ( d> 100 *μ*m, in red) while very small droplets ( d< 10 *μ*m) remain airborne and are transported by the puff is not surprising. What is interesting to note, however, is that droplets of intermediate sizes between 10 and 100 *μ*m can display either behavior. This can be clearly seen in Fig. 5(a), which shows the deposition side of the typical Wells' curve. At approximately 75 *μ*m, for example, the settling time varied roughly between 30 and 60 s, that is, up to 30% different than the value predicted by a low-order model^5^ where the motion, turbulence, and humidity content of the puff were neglected. It is expected that much higher variations would have been observed for smaller droplets, should longer simulations times had been performed. Despite such large variations in settling times, the evolution of the *total* mass of the droplet cloud (i.e., all droplets in the air) remains fairly similar to the behavior described by the one-dimensional (1D) model.^5^ The variation with time of the total suspended mass, *m*, normalized by the initial liquid mass *m*_0_ is shown in Fig. 5(b) for all the realizations. Small differences of $m/m_{0}$ over time are evident from realization to realization and in relation to the estimate without any flow information^5^ (shown as a solid red line).

**FIG. 6.**
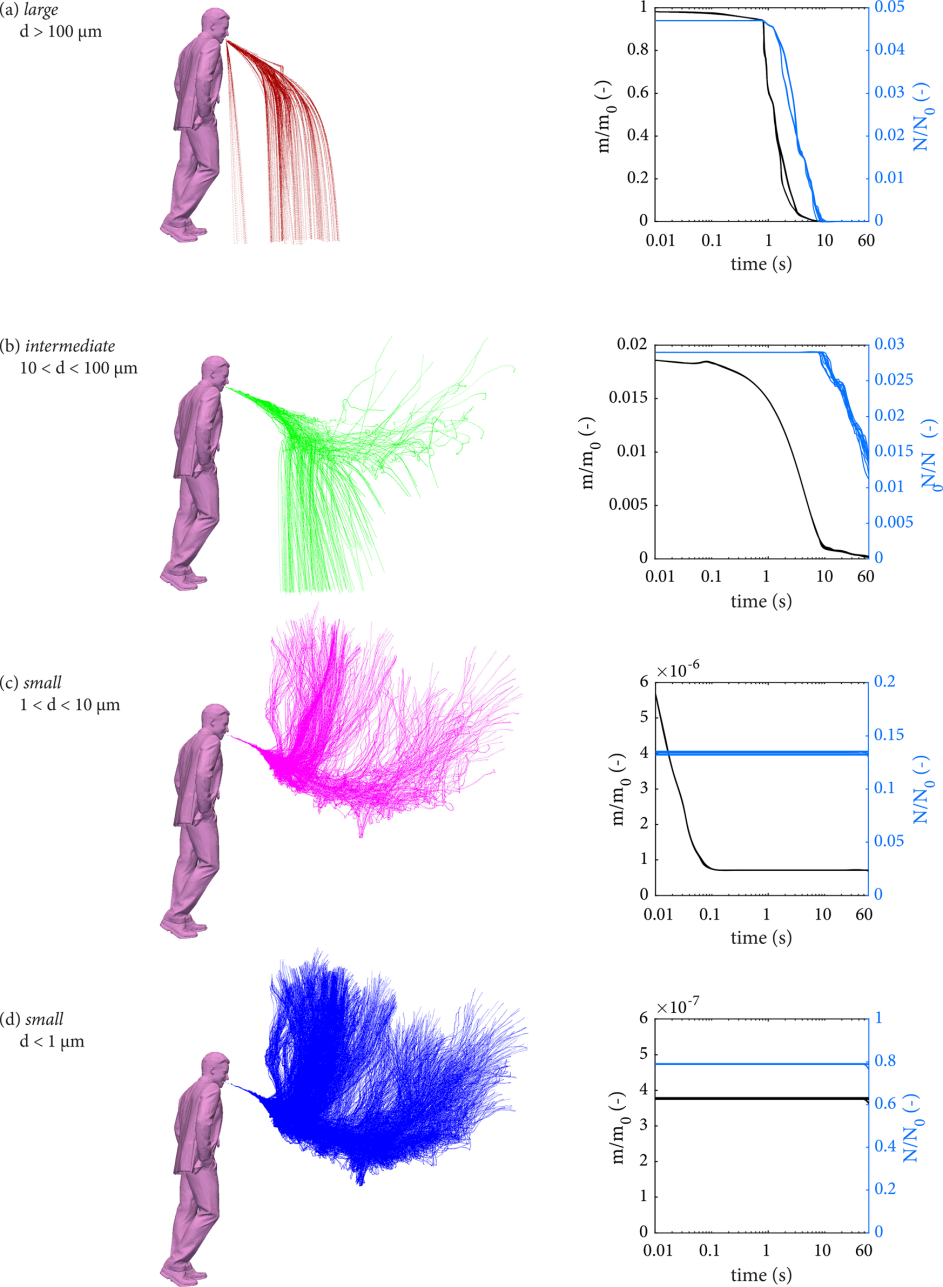
Analysis of droplet classes: (a) large, (b) intermediate, (c) small (1 *μ*m <d< 10 *μ*m), and (d) very small ( d< 1 *μ*m), for all the realizations. Droplet trajectories and temporal evolution of suspended number of droplets and suspended mass normalized by the respective initial values.

The characteristic behavior of each droplet size class is then analyzed in terms of ensemble quantities in a single realization. Based on their initial diameters once exhaled, Fig. 6 shows large droplets ( d> 100 *μ*m), intermediate size droplets (10 *μ*m < d< 100 *μ*m), and small droplets (1 *μ*m < d< 10 *μ*m and d< 1 *μ*m), from top to bottom, respectively. On the left, the trajectories of the droplets in each category are shown, accompanied by the cloud's respective normalized number $N/N_{0}$ and mass $m/m_{0}$ (*N*_0_ and *M*_0_ are the initial values at the ejection point). Most of the mass exhaled, up to 93% of the total, is contained in scarce large droplets and promptly removed by gravity within the first few seconds, as described by de Oliveira *et al.*^5^ After this time, most droplets suspended are small (colored blue and pink) and follow the gas flow, while their ensemble quantities are unaffected up to 60 s. Nonetheless, 1.5% of the total droplets emitted remains suspended as droplets of intermediate size, which account for 10% of the total mass of liquid emitted—that amount is roughly one thousand times the mass contained in small droplets. The behavior of the intermediate-size droplets is particularly interesting, varying between ballistic and airborne/aerosol behavior, discussed in detail next.

**FIG. 7.**
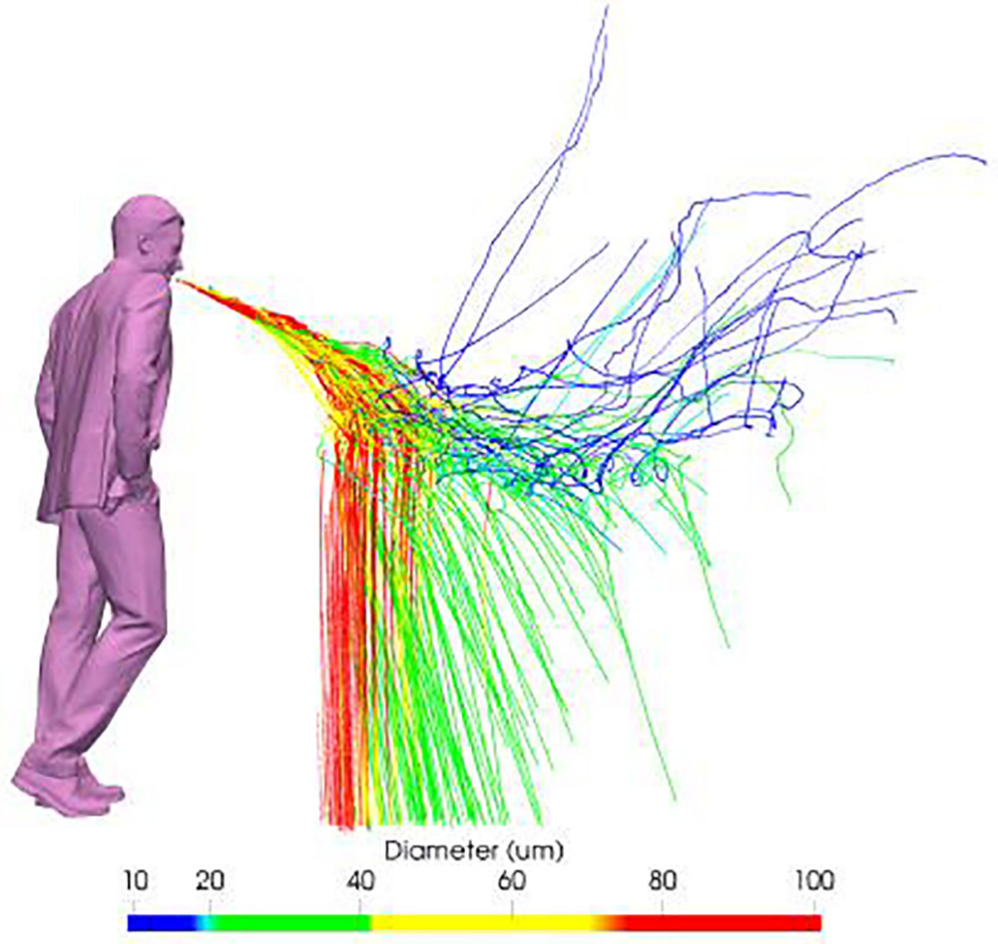
Visualization of trajectory of the droplets in the range 100 *μ*m <d< 10 *μ*m, colored by their initial diameter.

The behavior of droplets with initial diameter in the range 10 *μ*m < d< 100 *μ*m is given in more detail in Fig. 7. Within this size range, a combination of ballistic behavior and airborne/aerosol behavior is observed. The droplets ∼100 *μ*m show similar ballistic behavior to their larger counterparts, while droplets ∼10 *μ*m exhibit pure airborne/aerosol behavior. Droplets with diameter between ∼20 and 70 *μ*m (yellow and green) are marked by an initial airborne behavior until falling out from the gas puff. This seems to be determined by a combination of the recirculating buoyancy-driven azimuthal motion and turbulence,^58^ leading to a continuous fall-out process with droplets. Thus, droplets of similar size may fall out near the source or be sustained for much longer horizontal distances without promptly bending upward as in the case of small droplets.

**FIG. 8.**
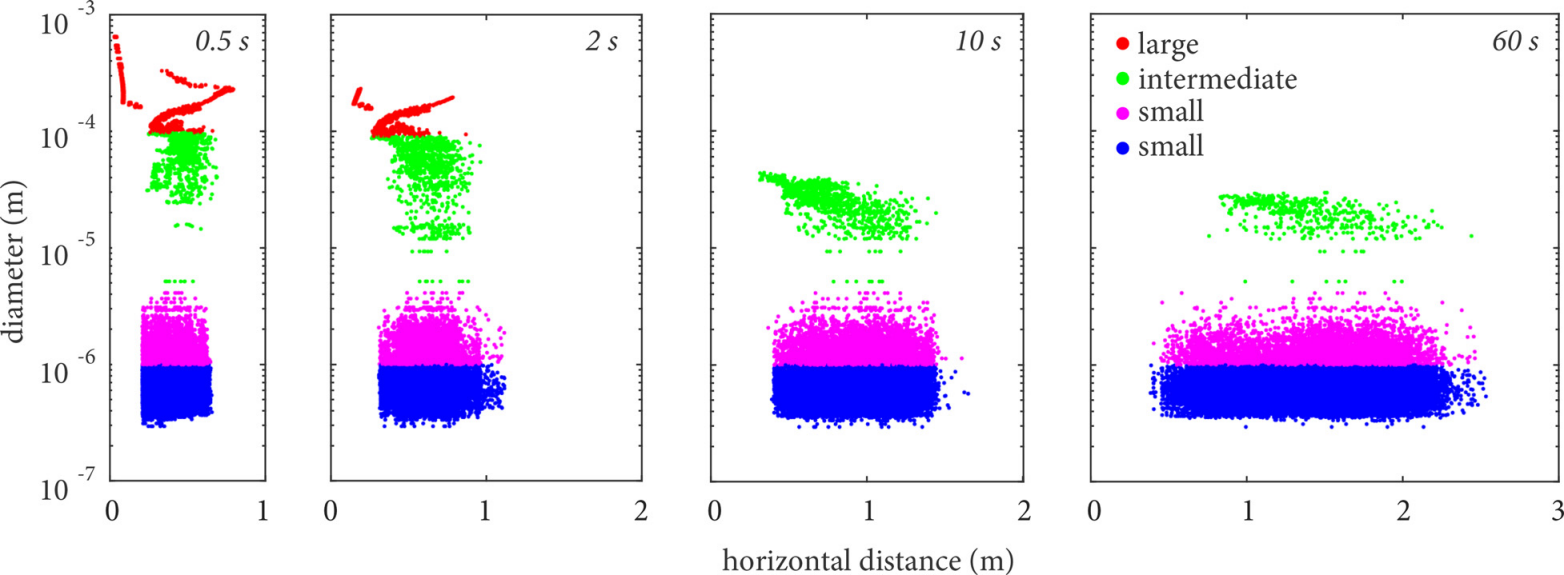
Scatter plots of instantaneous droplet diameter vs droplet horizontal distance from the emitter for all realizations, colored by different size category based on their initial diameter.

The spread of suspended droplets in the respiratory puff is quantified in Fig. 8, which shows the scatter plots of droplet position and their corresponding sizes at 0.5, 2, 10, and 60 s after the start of the cough.

The results not only confirm the previous discussion, but reveal the great variability associated with the spread of the droplets in the horizontal direction. Within one meter from the emission, the advancement of a droplet front comprising large- to intermediate-sized droplets can be observed. In parallel, the spread of a droplet cloud composed mainly of droplets of initial size below 10 *μ*m occurs in the horizontal direction, quickly reaching 0.5 m within 2 s after the cough and spreading all the way to 2.5 m after 60 s. As discussed next, most of this variability actually results from differences between events, in addition to the continuous fall-out process in a single cough.

**FIG. 9.**
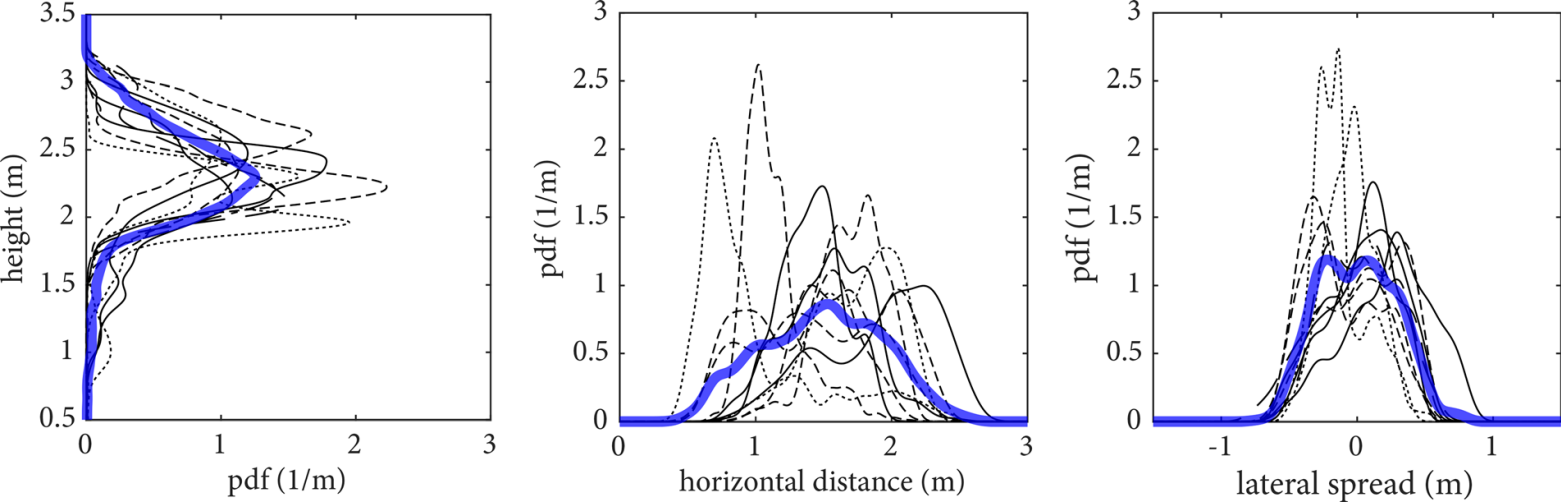
Probability density functions (pdfs) of the position of all the suspended droplets at 60 s after the cough. The black lines correspond to the results of each single realizations whereas the thick blue line is the average of all the realizations.

Figure 9 shows the probability density functions (pdfs) of the position of all suspended droplets at 60 s, using (a) data from each individual realizations, shown by black lines, and (b) data from all the realizations, shown by blue lines. The spread in the horizontal direction is noticeably larger than that in any other direction. A strong variability can be seen between the realizations, as each horizontal pdf is characterized by a peak located at a distinct distance from the emitter, which can range from 0.5 to 2.5 m. In fact, droplets in some cough realizations do not reach a 2 m distance, while for other realizations a significant amount can be present as much as 2.5 m away from the emitter. This shows the importance of turbulence and its associated stochasticity in carrying the suspended droplets over large distances. In the vertical direction, the droplets tend to concentrate at around ≈2.25 m, at 60 s from emission, as a consequence of buoyancy. The variability in the lateral spread is only caused by the turbulence from the cough. These results highlight that while a 1D model is capable of accurately modeling the total suspended mass evolution of the droplets (as shown in Fig. 5), the spatial distribution of droplets varies considerably in each realization, indicating that turbulence needs to be accounted for, if one intends to estimate droplets spatial spread. Furthermore, the present results are collected under the nearly stagnant ambient conditions considered in this analysis, but in the presence of initial momentum in some direction, the spread of the suspended droplets could easily change as in the case of the Guangzhou restaurant outbreak.^59,60^

**FIG. 10.**
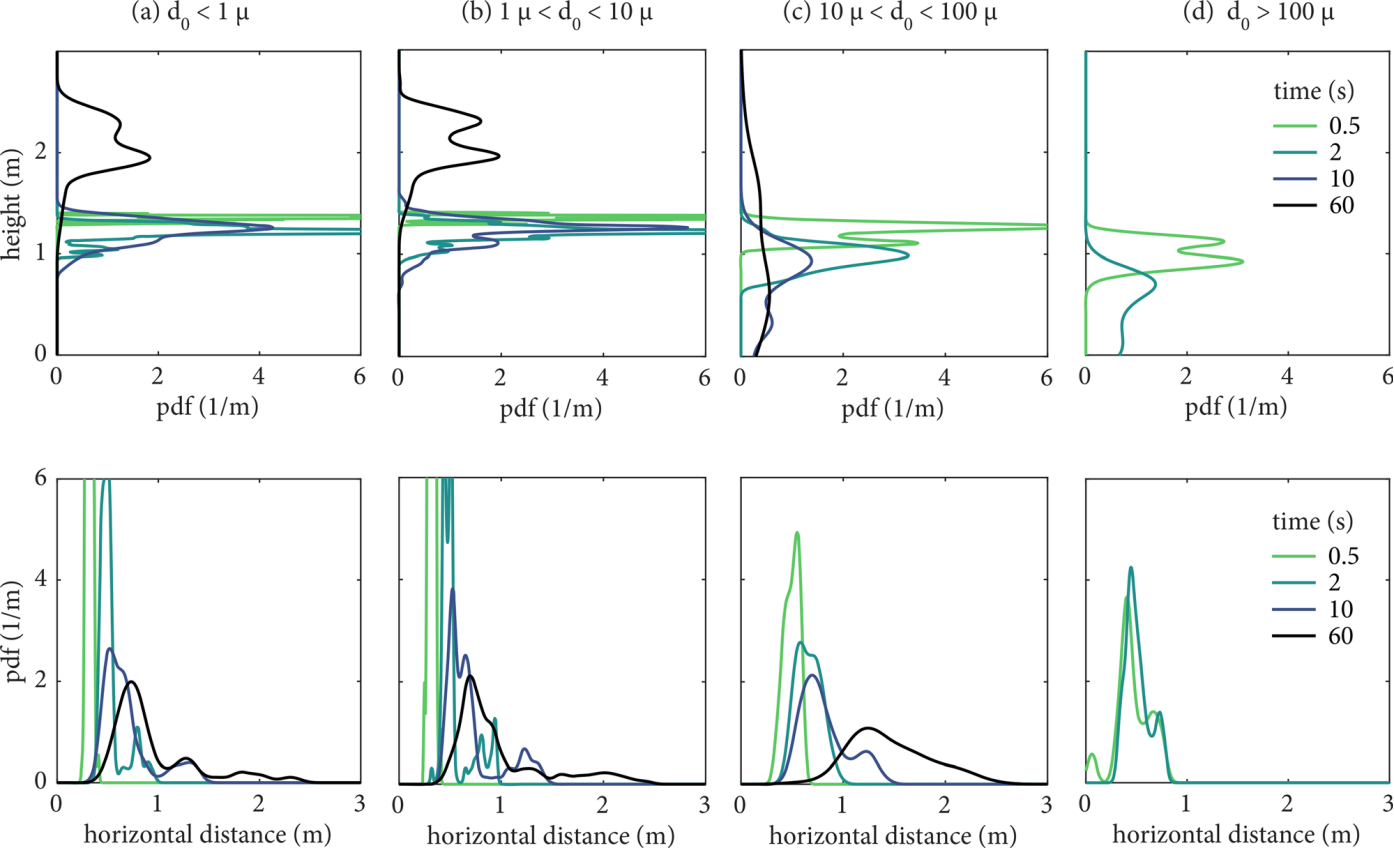
Probability density functions (pdfs) of the position of the suspended droplets at different times after the emission. The droplets are separated according to their initial size category. The results are compiled from all the cough realizations. (a) d_0_ < 1 *μ*m, (b) 1 *μ*m < d_0_ < 10 *μ*m, (c) 10 *μ*m < d_0_ < 100 *μ*m, and (d) d_0_ > 100 *μ*m.

The buoyancy-induced bending of the jet clearly affects the horizontal and vertical displacement of droplets, depending on their initial size category. As shown in Fig. 10, which provides the pdfs of the position of the suspended droplets compiled over all realizations for different droplet initial size categories, the small droplets are carried away from head height after 60 s, while they are concentrated mostly within 1 m from the emission source in the horizontal direction. In contrast, intermediate-sized droplets are sustained at head height until 10 s and spread at various heights at 60 s. Additionally, due to their large momentum, 10–100 *μ*m droplets can reach long horizontal distances, being mostly located between 1 and 2 m after 60 s. The very large ones (>100 *μ*m) have disappeared from the ensemble by 10 s due to settling. Therefore, the combination of (i) ballistic motion and gravitational settling for the large droplets, (ii) small droplets following closely the gas flow, (iii) intermediate size droplets showing both behaviors, and (iv) position pdfs that are wide, it is evident that potentially virus-carrying liquid can be found in large regions in space.

In the context of risk, as it will be shown in Sec. IV, although droplets in the 10–100 *μ*m size range are few in number, their originally large volume means they might carry a significantly larger number of pathogens than droplets typically classified as aerosol/airborne (<10 *μ*m). Those droplets can be easily inhaled by humans,^13^ and their viral content might be sufficient to lead to infection if inhaled depending on the concentration of pathogens in the respiratory fluid and additional factors.^61^ Here, we suggest that this size category cannot be directly classified as pure airborne or pure ballistic, and that their role in transmission, both short and long-range airborne transmission, cannot be overlooked.

## IV. DISCUSSION: CONSIDERATIONS FOR PHYSICAL DISTANCING

In this section, the results are put in the context of short-range airborne transmission, illustrating how the flow-driven stochasticity inherent to a cough impacts on the viral content potentially inhaled by a susceptible individual. The inhalation of virus-laden droplets and aerosols is idealized as the process of “probing” air from the breathing zone, represented as a 0.2-m spherical control volume (see Sec. II B) from which a total amount of virus inhaled over a time *t*^′^ from the beginning of the cough is given as:

$$N_{v,S}(t\prime )=\int_{0}^{t^{\prime }}\frac{N_{v,bz}(t)}{V_{bz}}{\dot{V}}_{b}dt,$$ (5)

where $N_{v,bz}$ is the instantaneous number of viral copies within the breathing zone volume *V_bz_*, and ${\dot{V}}_{b}$ is the average breathing rate. One should note that a number of other flow processes occurring in the vicinity of the susceptible individual are not considered in such an approach, such as near-field buoyancy-driven flows or the inhalation flow itself around the mouth and nose. A homogeneous concentration of virus in the respiratory fluid is assumed across all droplet sizes; hence, the stochasticity related to the presence or not of virus in small droplets^62^ is also not considered. Therefore, the present discussion helps assess the flow-induced stochasticity in isolation and not the randomness in virus exposure associated with other phenomena.

**FIG. 11.**
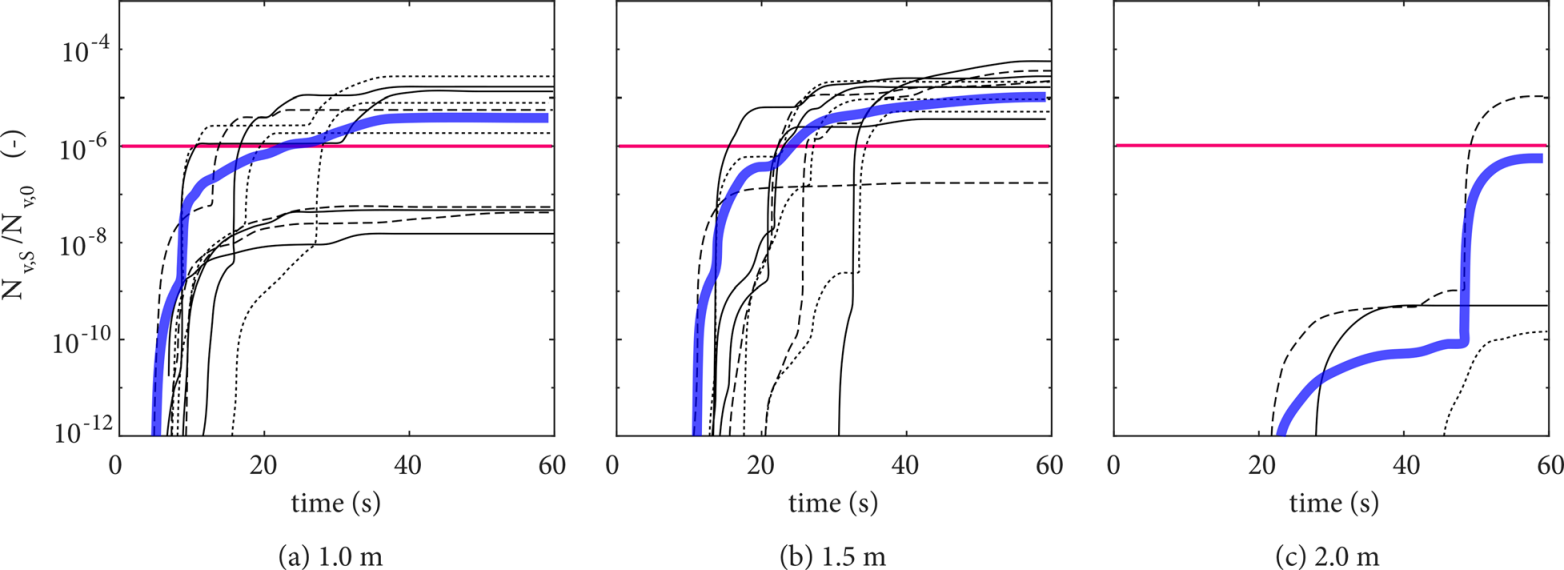
Number of virus N_*v*,*S*_ potentially inhaled by a receptor at horizontal distances 1.0 (a), 1.5 (b), and 2.0 m (c) from the mouth of an infectious individual. Values are normalized by the initial number of virus copies $N_{v,0}$. The red line corresponds to a single virion inhaled if a viable viral load at the mouth of 10^9^ copies/ml of respiratory fluid is considered.

The evolution of the number of potentially inhaled viral copies in each realization is given in Fig. 11, evaluated at horizontal distances of (a) 1.0, (b) 1.5, and (c) 2.0 m from the mouth of the infectious individual, and an ensemble average (blue line) of all events is also provided. The results are given normalized in terms of the total amount of viable viral copies emitted in a single cough, N_*v*,0_. For reference, this value is ( $1.7\times 10^{-3})\times V_{l}$, where $V_{l}$ is the viable viral load at the mouth (given in copies of viable virus per ml of respiratory liquid). For example, an $N_{v,S}/N_{v,0}$ value of $10^{-6}$ shown in the y axis (red line, Fig. 11) would roughly correspond to one single viable virus if a viral load of 10^9^ copies/ml of respiratory liquid at the mouth is considered—which is typical of symptomatic individuals at the onset of the symptoms for SARS-CoV-2^63^—while a value of $10^{-9}$ would correspond to a single virus if a 1000 times higher viral load is considered instead (i.e., 10^12^ copies/ml), which could be the case for more infectious variants of the SARS-CoV-2 virus.^64^ Due to the assumption of homogeneous distribution of virus over all droplet sizes as well as the spatially averaged concentration of virus in the breathing zone [ $N_{v,bz}/V_{bz}$, Eq. (5)], a value of $N_{v,S}$ lower than one would appear depending on the viral load assumed, which should be disregarded when interpreting the results.

Overall, the high degree of inhomogeneity in the droplet field is reflected in terms of the number of potentially inhaled viral copies, which is observed to vary both in terms of the axial location of the probe [Figs. 11(a)–11(c)] and from event to event (each line in the plots). Considering the present results in light of a viral load of 10^9^ copies/ml, sufficiently high levels to cause the disease (between 10 and 100 virions^65^) would correspond to $N_{v,S}/N_{v,0}$ values between $10^{-5}$ and $10^{-4}$. These are reached as fast as 20 s after a cough at distances between 1 and 1.5 m from the emitter, respectively.

In addition to the differences between how fast significant viral content may reach a susceptible individual between each cough, what can also be noticed from the results in Fig. 11 is the high variability of the final amount of virus potentially inhaled. After 60 s from a cough and closer to the emitter, at 1-m distance, almost 5 orders of magnitude difference in viral content is found between minimum and maximum values. Such differences are associated with the polydispersed nature of the droplet cloud emitted, as scarce large droplets carrying a high amount of viral content (i.e., 10 *μ*m <d< 100 *μ*m) only occasionally appear in the breathing zone. At 1.5-m distance, less variation is found and $N_{v,S}/N_{v,0}$ values are somewhat higher than at those at 1.0 m, which is associated with the onset of buoyancy effects at such distance following the jet-dominated region close to the emitter, causing a net updraft of the droplet cloud, as discussed previously.

**FIG. 12.**
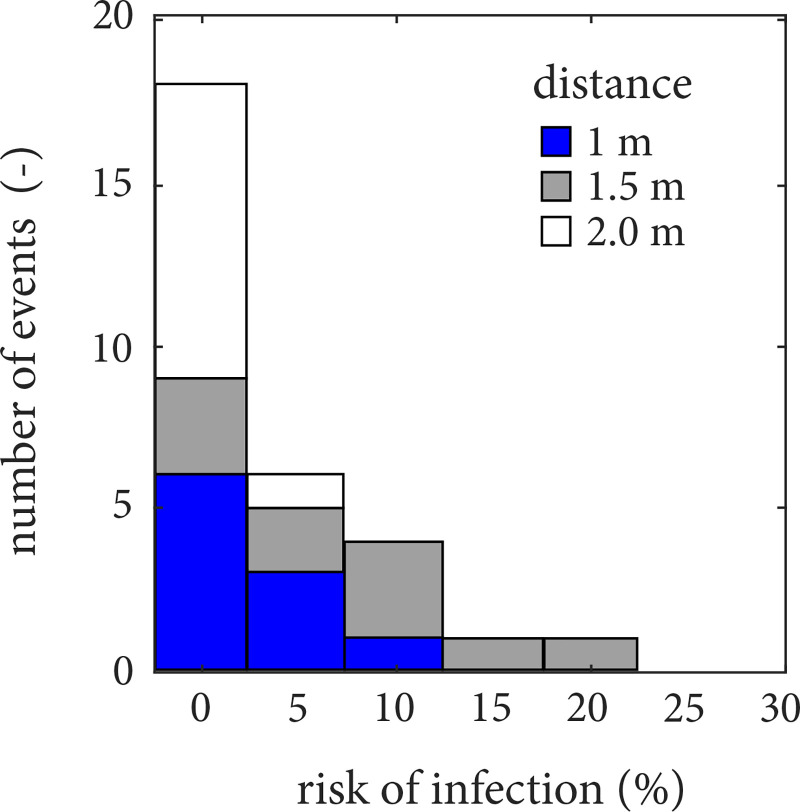
Histograms of risk of infection at 1 m (blue bars), 1.5 m (grey bars), and 2.0 m (white bars) distance from the infectious emitter, compiled by estimating the risk for each cough event. Bars of each individual histogram are placed on top of each other. The risk has been calculated according to a dose-response risk model^65^ for a viable viral load at the mouth of 10^9^ copies/ml of respiratory fluid.

Such large variations of $N_{v,S}/N_{v,0}$ are also found to be translated to large variations of risk of infection. By considering a dose-response model^65^ used in a previous work by some of the present authors,^5^ the values of $N_{v,S}$ at 60 s from emission were used to evaluate the corresponding risk of infection at distances 1, 1.5, and 2.0 m from the emitter. These results are given in Fig. 12 considering a viral load of 10^9^ copies/ml for illustration; one should note that such risk values are, of course, highly dependent on the viral load assumed. As shown in Fig. 12, cough events at 2 m from the source appeared “mostly safe” up to 60 s from emission, while at 1.5 m distance significant risk was observed, ranging from 1 to 20%. Interestingly, in this particular case, lower risk was observed at 1 m in relation to 1.5 m, as the subject coughs downward and the buoyancy-driven effects discussed previously are responsible to bring the emitted particles upward to face level around the horizontal distance of 1.5 m. Furthermore, as a way to demonstrate the importance of considering the statistics, if one uses the ensemble-averaged droplet distributions at the breathing zone at 1.5 m [blue curve, Fig. 11(b)], one gets a risk of 4% at 60 s, while the true average risk from the histograms of Fig. 12 (grey bars) at the same location and exposure time is 8%. This large difference is due to the non-linearity associated with the connection between risk and dosage, showing that knowledge of the statistics is vital for the accurate estimation of the transmission risk.

At a 2-m distance from the emitter, three out of the ten events exhibited non-zero virus in the breathing zone, i.e., non-zero risk infection, as shown in Fig. 11. In particular, one of the cases shows a significant increase in N_*v*,*S*_ around 50 s as one of the scarce mid-sized droplets (i.e., 10 *μ*m <d< 100 *μ*m) entered the breathing zone, causing a sharp rise in $N_{v,S}/N_{v,0}$ from $10^{-9}$ to $10^{-5}$, which in terms of risk of infection translates to roughly 5% for a viral load of 10^9^ copies/ml (Fig. 12). Note that the number of virus copies in the breathing sphere will not only be altered by flow and ambient conditions,^44,45^ but also because the viral load can be as high as 10^12^ copies/ml in severely affected individuals.^64^ This can lead to a significant risk of infection at over 1-m distance. This particular event demonstrates well the stochastic nature of short-range transmission and the often neglected risk associated with wandering mid-sized droplets. Even if simple low-order models suggest that most of such droplets settle by gravity within the vicinity of the emitter,^66–68^ it is possible that a combination of flow/turbulence-driven events lifts a single droplet for long distances which, if inhaled, is likely to cause the disease. Such “unlikely” events become especially relevant when one considers that a sick, infectious individual may cough very frequently throughout the day. Therefore, it is not only essential to account for turbulence/flow effects to obtain an accurate representation of the transport of droplets/aerosols in the near field of an infectious individual, as it has been recently brought forward here and in recent works,^25–27,30^ but, most importantly, the process should be treated from a statistical perspective considering its inherent stochasticity as demonstrated in this paper. This way, more accurate risk of infection models can be derived from calculations, such as those presented in this work, to define mitigation measures, such as physical distancing in the context of SARS-CoV-2 and its variants or other airborne pathogens.

## V. CONCLUSIONS

In this work, the stochasticity of the flow associated with a cough and its impact on short-range droplet distribution and, by consequence, disease transmission is discussed. Ten LES realizations of a cough were performed to capture the flow dynamics and spread of respiratory droplet clouds. The gas flow evolution was first presented with the help of spatial distribution of a passive tracer, defined as unity at the mouth and zero in the ambience. The flow was initially exhaled as a turbulent jet and subsequently became a floating puff, consistent with the description of Bourouiba *et al.*^2^ The trajectory of the droplets was analyzed using the Lagrangian tracking method considering local temperature, relative humidity, gravity, and local turbulence effects. By looking at the trajectories, the intermediate-sized droplets exhibited an unexpected behavior, in that some droplets with an initial diameter up to 75 *μ*m remained suspended within the puff, traveling horizontal distances of over 2.0 m within 60 s. Thus, it is unclear if a 2.0 m distance is safe to be practiced even outdoors, as these droplets may carry a significantly large amount of virus over large distances. In the case of a cough within stagnant air, that is, in the absence of wind and ventilation-driven streams, the Wells^11^ size definition of ≈100 *μ*m seems to apply well to distinguish large droplets ( d> 100 *μ*m) exhibiting a ballistic behavior from those smaller droplets that may remain suspended in air and follow the turbulent puff for a long duration. Very small droplets, viz. d< 1 *μ*m and 1 *μ*m < d< 10 *μ*m behaved identically, always following the gaseous flow. Alternatively, if a size cutoff of 10 *μ*m is used instead, we show that this may underestimate both short-range and long-range transmission.

These results were compared with previous analyses using quiescent air without turbulence.^5^ It was found that the total suspended mass of the droplet cloud was in good agreement with the one-dimensional quiescent-air analysis for the duration of the event (i.e., 60 s). Differences of up to 2–3 times between the mass of the droplet cloud and the value predicted by de Oliveira *et al.*^5^ were observed at later times (>10 s), mostly due to the effect of turbulence on droplets in the intermediate 10–100 *μ*m size range, which cannot be captured by the 1D modeling.

Finally, the main impact of turbulence was found on the spatial distribution of the droplet cloud. The spread of the droplets exhibited a strong variability with horizontal distance: some realizations showed few droplets over a 2 m distance, whereas others had a significant number of droplets at the 2 m mark. This effect had great impact on the viral content inhaled by a susceptible individual away from the emitter. Differences in the number of inhaled virus copies can vary by several orders of magnitudes between realizations. At very high concentration of viral load (10^12^ copies/ml), a significant risk of infection can be present at over 1 m distance after 60 s for a single cough.

The individual realizations and droplet trajectories were used to estimate the risk of disease transmission for each cough event with a dose-response model. It was found that each cough event has a different transmission potential and that significant fluctuations in the risk are found at all distances. Thus, the mathematical models typically used for developing physical distancing guidelines must include the inherent variability typical of the flow associated with a cough.

## Data Availability

The data that support the findings of this study are available from the corresponding author upon reasonable request.
